# The dynamics of a family’s gut microbiota reveal variations on a theme

**DOI:** 10.1186/2049-2618-2-25

**Published:** 2014-07-21

**Authors:** Patrick D Schloss, Kathryn D Iverson, Joseph F Petrosino, Sarah J Schloss

**Affiliations:** 1 Department of Microbiology and Immunology, University of Michigan, 1520A Medical Science Research Building I, 1150 W. Medical Center Drive, Ann Arbor, MI 48109, USA; 2 Department of Molecular Virology & Microbiology, Houston, TX 77030, USA

**Keywords:** Microbiome, Family, Feces, Dynamics, Chronosequence, Community, Development

## Abstract

**Background:**

It is clear that the structure and function of the human microbiota has significant impact on maintenance of health and yet the factors that give rise to an adult microbiota are poorly understood. A combination of genetics, diet, environment, and life history are all thought to impact the development of the gut microbiome. Here we study a chronosequence of the gut microbiota found in eight individuals from a family consisting of two parents and six children ranging in age from two months to ten years old.

**Results:**

Using 16S rRNA gene and metagenomic shotgun sequence data, it was possible to distinguish the family from a cohort of normal individuals living in the same geographic region and to differentiate each family member. Interestingly, there was a significant core membership to the family members’ microbiota where the abundance of this core accounted for the differences between individuals. It was clear that the introduction of solids represents a significant transition in the development of a mature microbiota. This transition was associated with increased diversity, decreased stability, and the colonization of significant abundances of Bacteroidetes and Clostridiales. Although the children and mother shared essentially the identical diet and environment, the children’s microbiotas were not significantly more similar to their mother than they were to their father.

**Conclusions:**

This analysis underscores the complex interactions that give rise to a personalized microbiota and suggests the value of studying families as a surrogate for longitudinal studies.

## Background

Numerous studies have identified associations between deviations in the gut microbiota (that is the community of microorganisms living within the gastrointestinal tract) and diseases as varied as psoriasis, diabetes, colon cancer, and susceptibility to *Clostridium difficile* infection [1-4]. The mechanisms that give rise to an individual’s microbiota as well as the deviations from their normal microbiota are poorly understood. In light of our growing appreciation for the role of the microbiota in maintaining a healthy state, with isolated exceptions such as fecal microbiota transplant as a treatment for recurrent *Clostridium difficile* infection [5], we are largely powerless to manipulate the microbiota to achieve long-term transitions to a healthy state. Fundamental to this problem are the sources of our microbiota and the relative importance of numerous variables that can affect the structure and function of the microbiota.

Genetics, environment, life history characteristics, and diet are expected to have significant long-term impact on the composition of one’s microbiota. Studies of monozygotic and dizygotic twins suggest that monozygotic twins who share an identical set of genes have more similar microbiotas than dizygotic twins who only share half their genes [6,7]; however, the biological significance of the difference in similarity is likely minimal. Furthermore, shared environments and diet confound the similarity between twins. In addition, the microbiotas of co-habiting individuals tend to be more similar than individuals that are not co-habiting; this argues for the importance of a shared environment and similar diet in shaping the microbiota [8]. Life history characteristics such as whether one was breastfed or bottle-fed or born vaginally or via Cesarean section have been shown to impact the immediate structure of the individual’s microbiota in infancy [9,10]; however, it is unclear what long-term impacts these characteristics have on the composition of the microbiota. One notable example of such investigations was a 2.3-year time course study of a child’s life starting at birth [11]. Discrete changes in his microbiota were associated with fever and coincident transitions in diet and antibiotic therapies. That study suggests that large perturbations are needed to shift a child’s microbiota from one community structure to another. Several other studies have employed antibiotic perturbations and observed that the structure of the microbiota largely returns to its pre-treatment state after the cessation of the treatment [12-14]. Similar results have been observed among individuals who undergo bowel preparation prior to colonoscopy [15]. In short-term diet perturbation studies, groups of individuals have been given diets that are discordant with their normal diet, and although their microbiota changes, it does not converge to resemble the microbiota of others receiving the same diet. In addition, when the individuals return to their normal diet, their microbiotas also return to their previous community structure [16,17]. These studies and numerous others indicate that the microbiota is relatively robust to perturbation as numerous studies have shown that the structure of an individual’s microbiota is more similar to itself over time than it is to the microbiota of another individual [6,18,19]. The model that emerges from these studies is that the fundamental source for one’s microbiota is the physical and biological environment in which the individual lives. Meanwhile, other factors including genetics, immunological exposures, environment, life history characteristics, diet, and overall philosophy to using antibiotics and other clinical interventions, sculpt the underlying community structure.

Families provide a unique platform for testing the factors that impact the membership and abundance of one’s microbiota because they provide greater opportunities to control for the factors that affect the structure of the microbiota. For example, an analysis of a family where one child becomes a vegetarian would improve our understanding of the effects of diet on the microbiota while controlling for the other factors. In addition, families with various-aged children may represent a chronosequence of the family’s microbiota [20]. Chronosequences could be used to understand how the microbiota develops over time without having to collect samples for numerous years from a single individual. They could also be useful as a tool for determining when an individual’s microbiota deviates from his or her siblings. Over short periods of time, analysis of a family’s microbiota could also inform our understanding of how perturbations to one individual’s microbiota would impact the microbiota of others in the family. These studies have been performed to understand the transmission of pathogens [21-23]. In light of these opportunities, we characterized the gut microbiota of a family with six children over the course of a month relative to a cohort of unrelated adults from the same geographic region.

## Methods

### Sample collection and DNA extraction

This study was approved by the University of Michigan Institutional Review Board. All subjects or their parents granted consent to participate in the study. The members of the family obtained fecal samples by scraping feces from toilet paper at their home and their place of employment using sterile wooden applicators [19]; the infant’s samples were obtained by scraping feces from his cloth diapers using sterile wooden applicators [11]. The parents obtained the samples for the children and kept a diary of the food the children ate during the course of the study. Because of the size of the family, it was not practical to record the amounts of each food consumed by the family members. Samples from unrelated adults in the broader community were collected from individuals residing in Ann Arbor, MI area (53 males, 102 females; ages 19 to 88 years). Subjects were excluded if they had had any signs of diarrhea in the previous seven days or were pregnant. All fecal samples were immediately stored at -20°C until DNA extraction. Total bacterial DNA was extracted from each fecal sample using the PowerSoil^®^-htp 96 Well Soil DNA Isolation Kit (MO BIO Laboratories Inc., Carlsbad, CA, USA) on an EpMotion 5075 liquid handling workstation (Eppendorf, Hauppauge, NY, USA).

### DNA sequencing and curation

The V3-V5 region of the 16S rRNA gene was amplified and sequenced using the 454 GS FLX pyrosequencing platform at the Baylor College of Medicine as described previously [24]. In parallel to the fecal samples, a mock community was included on each sequencing run for calculating sequencing error rates after curation [25]. All 16S rRNA gene sequences were curated using the mothur software package as previously described [25,26] and resulted in a final error rate of 0.009%. Sequences were clustered into operational taxonomic units (OTUs) using a 3% distance cutoff with the average neighbor clustering algorithm [27]. Taxonomic assignments were determined using a naïve Bayesian classifier trained using the RDP training set with an 80% bootstrap confidence threshold [28]. All samples were rarefied to 1,827 sequences per sample to avoid the detrimental effects of uneven sampling.

### Metagenomic shotgun sequencing and curation

For each of the eight family members, samples were collected at days 1, 15, and 26, which corresponded to the beginning, middle, and end of the study. Random genomic DNA from these samples was sequenced as previously described at the Baylor College of Medicine [29]. SeqPrep was used to remove primer sequences from reads (https://github.com/jstjohn/SeqPrep). All reads were pooled together and normalized using khmer’s digital normalization pipeline [30]. This excluded from assembly any read that had a median k-mer of length 20 that had previously been encountered at least 20 times. The excluded reads were saved for downstream analysis. The remaining reads were filtered by abundance, removing any low abundance and unique k-mers. The filtered reads were assembled by velvet with k-mer lengths of 31 and 35 [31]. The contigs from these assemblies were combined and de-replicated at 99% with CD-HIT [32]. The combined, de-replicated contigs were merged with minimus2 [33]. Merged contigs and singletons from minimus2 were screened by BLASTn for hits to human sequences. Contigs with hits to human sequences and the associated reads were removed from further analysis. Open reading frames (ORFs) were predicted from assembled contigs with MetaGeneAnnotator [34]. Gene counts were obtained by mapping reads to the predicted genes with bowtie [35]. UBLAST was used to assign each translated ORF to Kyoto Encyclopedia of Genes and Genomes (KEGG) orthology categories (KO) [36,37]. Those ORFs that mapped to genes that were not already assigned to a KO category or lacked a significant match in the KEGG database were pooled into a single category. To assign ORFs to operational protein families (OPFs), we first performed a database-independent all-versus-all BLASTP search of the ORFs. The resulting BLAST scores were used to calculate distances between the ORFs, which were clustered using the average neighbor clustering algorithm with a 25% dissimilarity cutoff [38]. All samples were rarefied to 1,207,904 sequences per sample (approximately 114 Mbp per sample) to avoid the detrimental effects of uneven sampling.

### Community analyses

The mothur software package was used to calculate the inverse Simpson alpha diversity index, the θ_YC_ measure of community structure, and non-metric dimensional scaling (NMDS) ordinations for both the 16S rRNA gene and metagenomic sequence data [26]. Random Forest analysis of the 16S rRNA gene sequence data was performed using the randomForest R package with 10,000 trees (http://cran.r-project.org/).

### Data availability

The 16S rRNA gene sequence data, metagenomic sequence data, and the associated MIMARKS spreadsheet are available online (http://www.mothur.org/FamilyStudy).

## Results and discussion

We studied an eight-member family to investigate the formation of the personalized microbiota (Table 1). The mother and father had lived together for more than eleven years and had six children (two females and four males) ranging in age between two months and ten years old. The family members were of typical health with no recent antibiotic usage and showed no signs of obvious illness during the month covered by this study. All of the children were exclusively breastfed for at least the first 6 months of life and were partly breastfed until they were between 18 and 30 months old; the 2-month-old was exclusively breastfed and the 2-year-old was also being breastfed in addition to eating solids at the time of this study. The children and mother shared nearly all of their meals together and the father generally ate dinners on weeknights and all meals at weekends with the rest of the family (Additional file 1: Table S1). The family lived in a rural environment with pets and livestock. Within this context, we obtained daily fecal samples from each member of the family over the course of 26 days that we used to sequence the V3-V5 region of the 16S rRNA gene.

**Table 1:** Descriptive characteristics of family members

**Subject**	**Sex**	**Weight (kg)**	**Height (cm)**	**BMI (%ile)**	**Number of OTUs in core microbiota**
Infant (0 years old)	Male	7.0	ND	NA (NA)	4
2 years old	Female	10.2	82	15.2 (17%)	21
4 years old	Male	22.2	107	19.4 (>99%)	26
6 years old	Male	24.0	117	17.5 (86%)	32
8 years old	Male	22.8	127	14.1 (8%)	27
10 years old	Female	42.7	153	18.2 (66%)	18
Mother	Female	72.5	164	26.8 (NA)	58
Father	Male	96.5	183	28.8 (NA)	22

NA, not applicable. Weights and heights were measured as of the first day of the study. The number of operational taxonomic units (OTUs) in the individual’s core microbiota was determined by identifying those OTUs that had a minimum relative abundance of at least 0.05% in 95% of the individual’s samples.

**Figure 1:**
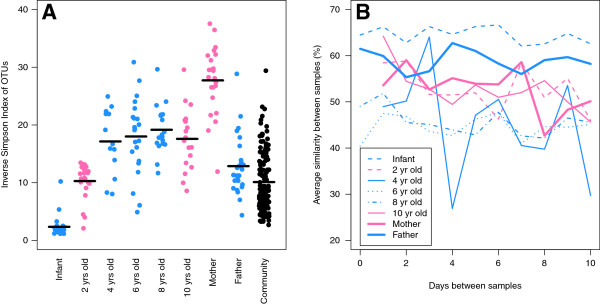
**Diversity and stability of the microbiota found within family members and individuals sampled from the broader community. (A)** Each point represents the inverse Simpson alpha diversity index for a sample collected from each individual. **(B)** The average similarity between samples collected from the same individual with varying number of days between when the samples were collected.

We measured the association between age and diversity and age and stability of each family member’s microbiota. Diversity was strongly associated with the degree to which the child was being breastfed (Figure 1A). The infant (exclusively breastfed) had the lowest diversity, the two-year-old (breastfed and eating solid food) had the next to lowest diversity, and the remaining four children (all weaned) had a similarly high level of diversity; the diversities of the three groups of children were significantly different from each other and the weaned children were not significantly different from each other. The mother and father each had diversities that were significantly different from each other and the children. Interestingly, the mother’s diversity was considerably higher than those observed within the family and among the Ann Arbor cohort and the father’s diversity was more similar to that of the Ann Arbor cohort. This is in contrast to previous observations that women who had recently given birth had lower diversity than normal women [39]. To assess whether these differences in diversity resulted in differences in the stability of each microbiota, we calculated the average β-diversity between an individual’s samples as a function of the number of days between their collection (Figure 1B). This analysis indicated that the infant had the most stable community and that the stabilities of the other family members were indistinguishable. Samples collected a day apart were just as similar as samples collected ten days apart. These data suggest that the transition from breast milk to solid foods brings about increased diversity and decreased stability in the gut microbiota.

**Figure 2:**
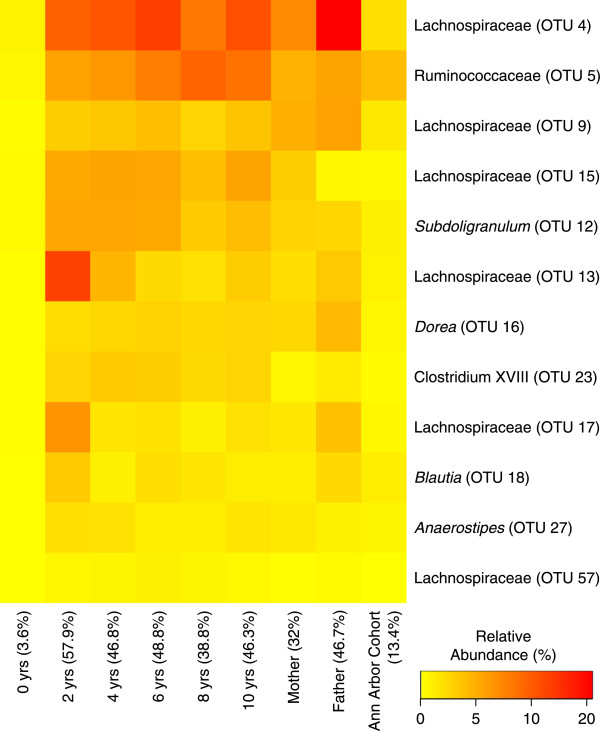
**Relative abundance of operational taxonomic units (OTUs) detected in all weaned individuals within the family.** The colors represent the average relative abundance of each OTU in each individual and as observed in the broader community. The percentages at the bottom of the heatmap indicate the percentage of sequences these OTUs represent in each individual.

It has been repeatedly shown that an individual’s microbiota is more similar to itself over time and more similar to family members than it is to unrelated individuals [6,18,19]. Thus, it was hypothesized that within the family there exists a ‘theme’ or core microbiota that distinguishes it from other families. To identify the core microbiota for each individual we identified those OTUs that had a relative abundance over 0.05% in at least 95% of their samples. This resulted in the identification of between 4 (infant) and 58 (mother) OTUs, which represented the core for each individual (Table 1); these represented between 1.8 and 7.7% of the OTUs that were detected for each individual. When we compared the lists of core OTUs from each individual to identify the family’s core microbiota, there was no overlap; however, when we removed the infant from the analysis, we identified 12 OTUs that were common to each person’s core microbiota (Figure 2). We also analyzed the Ann Arbor cohort and identified four OTUs that were found in 95% of the cohort members. These OTUs affiliated with members of the genus *Bacteroides* (OTU 3), family Lachnospiraceae (OTUs 4 and 9), and genus *Subdoligranulum* (OTU 12); OTUs 4, 9, and 12 were shared with the family core microbiota. The 12 OTUs that comprised the family’s core microbiota represented between 32.0 and 57.9% of the sequences in the 7 family members who were eating solids, 3.6% of the sequences in the infant, and 13.4% of the sequences obtained from the Ann Arbor cohort (Figure 2). These data indicate that a considerable fraction of each individual’s microbiota is represented by a core microbiota consisting of anaerobic Gram-positive spore formers.

**Figure 3:**
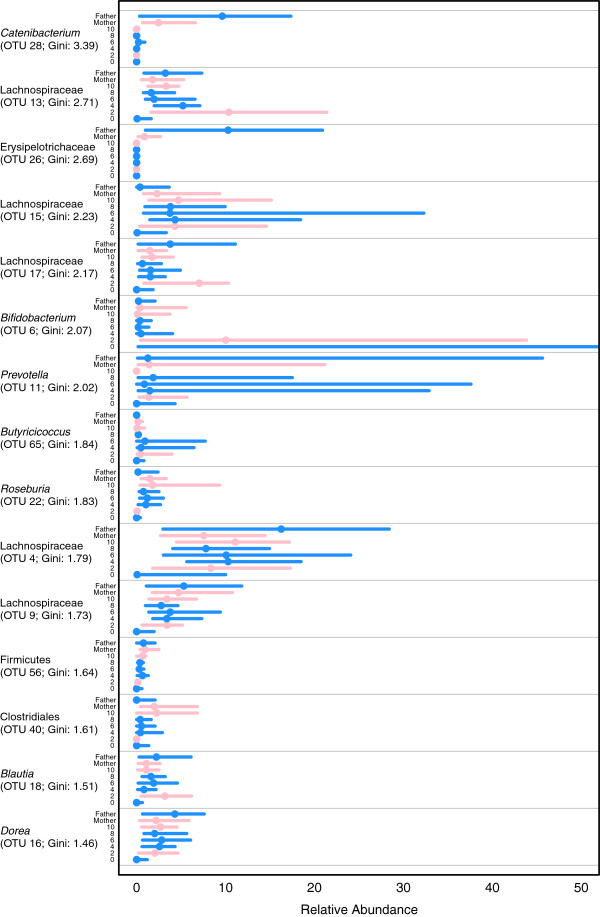
**The relative abundance of the 15 most important operational taxonomic units** (**OTUs) for distinguishing between family members.** OTUs are ranked by the Gini index as determined by using the Random Forest algorithm trained to distinguish between family members. Each line represents the range of relative abundances observed for each OTU across individuals. The solid dot represents the median relative abundance for that OTU in that individual.

Having identified the family’s core microbiota, we next attempted to identify variations on that theme within the family. We used the Random Forest machine-learning algorithm to identify OTUs that would allow us to distinguish between family members and obtained an out-of-bag error rate of 3.6%; at most, one sample from each individual was misclassified. When we limited the features to the top 15 OTUs that had the highest Gini index (Figure 3), the error rate was 7.2%. The low classification error rate indicated that each individual had a unique microbiota. The most obvious distinguishing OTUs included one affiliated with *Catenibacterium* (OTU 28), which had a high relative abundance in the parents and was most abundant in the father. The overall similarity of the parents’ microbiota is striking, as they are unrelated and spent more than 20 years apart prior to meeting. In spite of this, they still had similar community membership, but distinct abundances of those OTUs. The infant and two-year-old, both still at least partially breastfed, had an OTU that was affiliated with the *Bifidobacterium* (OTU 6). Although it was not one of the 15 strongest features, an OTU affiliated with the family Enterobacteriaceae (OTU 8; Gini: 1.53; median relative abundance: 24.5%) was also disproportionately high in the infant. The variations between the OTUs that distinguished the weaned children was more subtle and indicated that the differences were not due to the incidence of specific OTUs but were instead defined by the specific relative abundances of multiple OTUs. Overall, the difference in the microbiota of each family member was largely due to differences in abundance, not membership. These data support the hypothesis that one’s microbiota becomes individualized from an early age.

**Figure 4:**
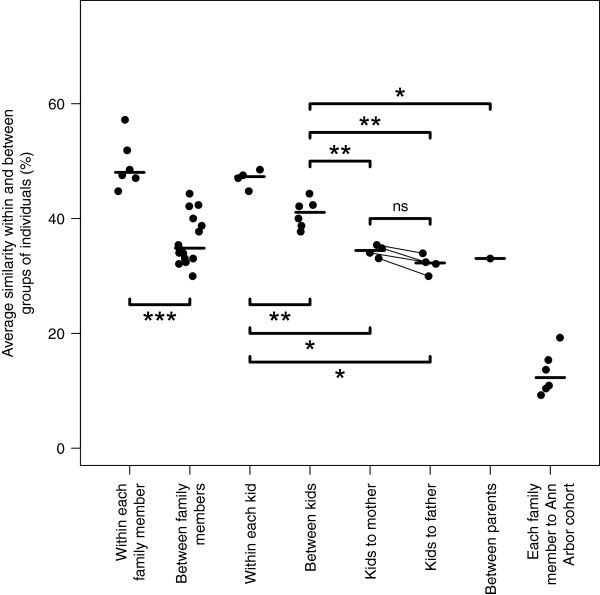
**Comparison of microbiota similarity within and between weaned family members and among members of the broader community.** Each point represents the average similarity for each individual within that comparison. Stars represent significance using non-parametric Wilcoxon test (*P < 0.05, **P < 0.01, ***P < 0.001). Lines drawn between points for similarity between the children and the mother and father indicate points that correspond to the same child. This comparison was not significant.

Given the large family size, it was possible to assess the relative importance of genetics and environment/diet on shaping the gut community structure using the children who had been weaned, their parents, and members of the Ann Arbor cohort (Figure 4). As expected, the community structure of each individual in the family was more similar to themselves than to any other family member (*P* < 0.001). Even though each of the children share 50% of their DNA with each other and their parents, the median similarity between children (θ_YC_ = 0.41) was significantly higher than their median similarity to either of their parents (θ_YC-Mother_ = 0.34; θ_YC-Father_ = 0.32; both *P* = 0.010); additionally, the children were more similar to each other than their mother and father were to each other (θ_YC-Parents_ = 0.33; *P* = 0.031). The similarity between each child and their mother was higher than to their father (Δθ_YC_ = 0.02); however, this difference was not statistically significant (*P* = 0.125) and not likely to be biologically significant. Interestingly, previous family-based studies have excluded the children’s father from the analysis [6,11]. This is notable when one considers that the children in this study were homeschooled by their mother with whom they share nearly all of their meals. These observations suggest that the father and his microbiota may be just as important as the mother in shaping a child’s microbiota. More broadly, it is likely that other caregivers and their environment may participate in shaping a child’s microbiota. Finally, the family members were as similar to each other as individuals from the Ann Arbor cohort were to each other (*P* = 0.433); however, based on the earlier Random Forest analysis, the family’s microbiota was clearly different from the broader community. The different microbiota represented within the family were clearly unique relative to each other and the broader community. The mechanisms that give rise to this uniqueness are likely a complex mixture of factors. Regardless, the family members appear to represent variations of a shared familial microbiota.

**Figure 5:**
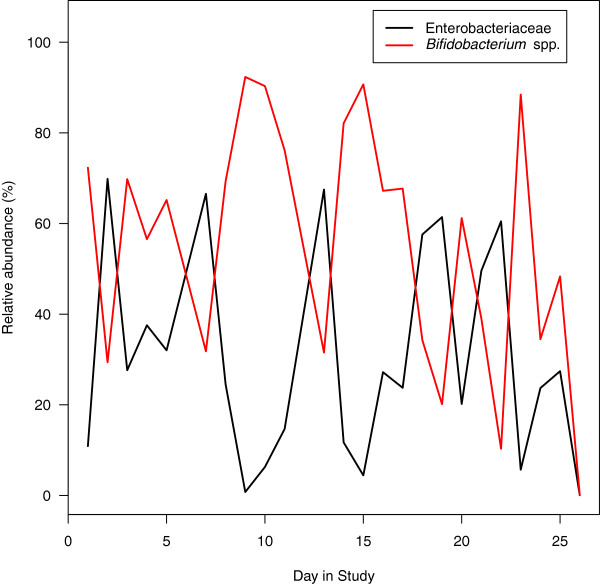
Dynamics of the dominant populations within the infant’s gut microbiota.

As the diversity data and Random Forest analysis suggested, the infant and two-year-old had the most distinct community profiles within the family. The dynamics of the infant’s microbiota was characterized by a series of transitions between single OTUs affiliated with the genera *Bifidobacterium* (median relative abundance: 63.2%) and *Escherichia* (median relative abundance: 25.9%; Figure 5). Such transitions are possibly a result of competition for resources between the two populations or predation by phage, which could suppress the size of the opposing population. Although the microbiota of the two-year-old was more similar to her weaned siblings, the same *Bifidobacterium*-related OTU that was found in her infant brother dominated her microbiota (median relative abundance: 10.0%). *Bifidobacterium* spp. have been associated with milk fermentation in breastfed infants [40]. This OTU was observed in all family members at lower relative abundance (median relative abundance: 0.3%; Figure 3). In the extreme case of transitioning from a diet consisting of only breast milk to solids, it was apparent that bacterial populations were being selected in response to the diet. Interestingly, the microbiota of the two-year-old represented a mixture of her exclusively breastfed brother and her weaned siblings.

**Figure 6:**
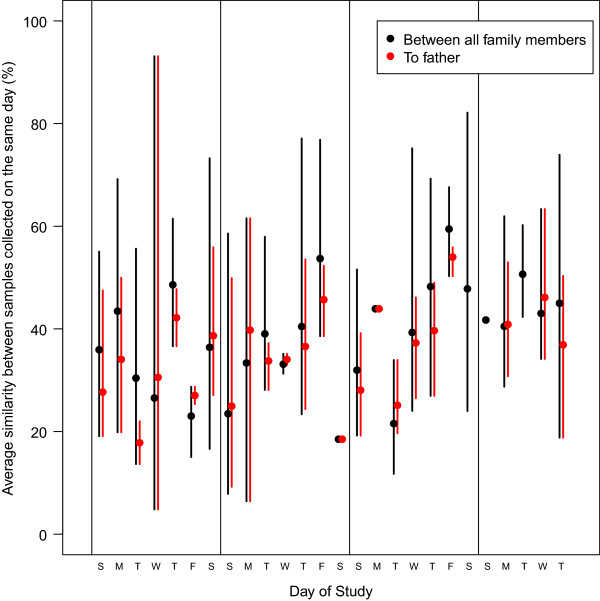
**Temporal dynamics of similarity between the weaned family members and between the weaned family members and the father.** The error bars represent the range between the minimum and maximum similarity among the family members in the comparison.

To assess the effect of diet on the structure of the gut microbiota, the parents recorded the food consumed by each individual in the family over the course of sampling (Additional file 1: Table S1). Because the quantity of individual foods was not recorded, we qualitatively assessed the family’s food record and identified samples collected the day after individuals consumed essentially the same diet. The distances between the communities in those samples were no more similar to each other than between samples collected on the same day from individuals who consumed different diets. On Saturdays and Sundays the father’s diet was more concordant with the rest of the family and he spent a greater amount of time with them. This led us to predict that following these days there would be periods of increased similarity in the microbiota between the father and the rest of the family. This was not observed (Figure 6). These results suggest that although the overall similarity in diet may make the family’s microbiota distinct from other individuals in the Ann Arbor community, the day-to-day variation in the family’s diet did not entirely explain the day-to-day variation in their microbiota. It is also possible that the differences in diet and environment were not large enough to elicit a significant response in the individuals’ microbiota. Rather, it may be that a shared environment and similar diet are long-term drivers of community structure and so day-to-day differences would not have a large impact on the individuals’ microbiota. Such a model depends on the existence of a stable microbiota with a sufficiently diverse community that is resilient to perturbations. Long-term tracking of such families will likely bracket perturbations of varying magnitudes (for example, illness, antibiotic usage, travel) and will allow us to better understand the forces that make an individual’s microbiota more similar or different from one another.

**Figure 7:**
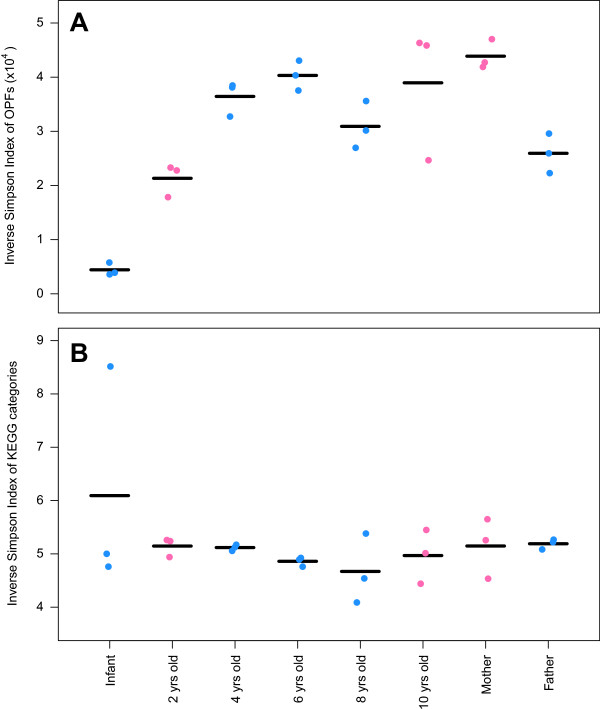
**The inverse Simpson alpha diversity index of the metagenome at three time points for each individual in the family using operational protein families (OPFs) (A) and Kyoto Encyclopedia of Genes and Genomes orthology (KEGG KO) categories (B).** The horizontal lines indicate the average diversity value for each individual.

**Figure 8:**
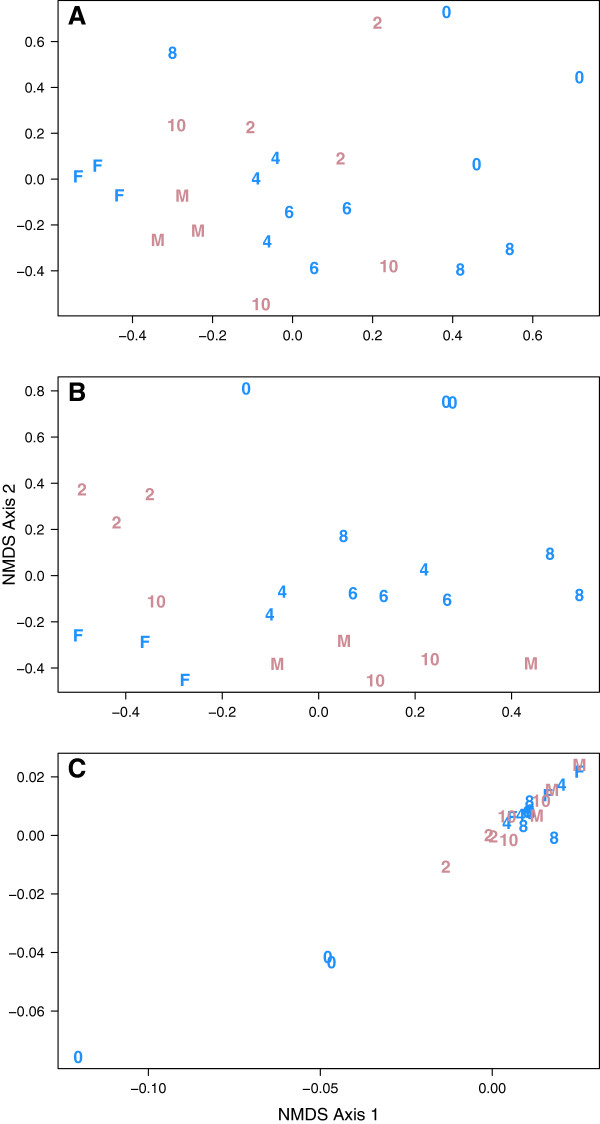
**Non-metric dimensional scaling ordinations of Θ**_**YC **_**distances between samples collected at beginning, middle and end of study that were analyzed based on operational taxonomic units (OTUs) (A), Kyoto Encyclopedia of Genes and Genomes orthology (KEGG KO) categories (B), and operational protein families (OPFs) (C).** The characters in the ordination correspond to the age or parental status of the individual and the color indicates their sex (female: pink; male: blue). The stress was 0.26 for the OTU-based ordination, 0.19 for the KEGG KO-based ordination, and 0.26 for the OPF-based ordination.

Finally, we assessed the genetic diversity of the family’s microbiota by performing shotgun metagenomic sequencing using samples collected from each individual at the beginning, middle, and end of the sampling period. Across all individuals, we observed a total of 4,499 KEGG categories and 675,908 OPFs. The inverse Simpson alpha diversity index calculated using OPFs (Figure 7A) followed a pattern that was similar to the diversity calculated using 16S rRNA gene sequence data (Figure 1A); no trends were observed when ORFs were assigned to KEGG categories (Figure 7B). When we assigned the ORFs to OPFs, 7.3% were shared across all family members, 36% were shared among the four weaned children, and 13% were shared between the two breastfed children. When we assigned ORFs to KEGG categories, 66% were shared across all family members, 77% were shared among the four weaned children, and 78% were shared between the two breastfed children. Whether we assigned the ORFs to clusters based on KEGG KOs or to OPFs, we were able to separate samples by individual, as there was a significant concordance between the taxonomic structure of the communities based on 16S rRNA gene sequences and the genetic structure of the communities based on both the KEGG KO and OPF data (R_OTU-KEGG_ = 0.58, R_OTU-OPF_ = 0.69; Figure 8). These results support the 16S rRNA gene-based analysis that the genetic composition is conserved between individuals, but that each individual has a unique microbiota.

## Conclusions

Our analysis of this family’s microbiota demonstrates that they represent a unique island within the possible permutations of microbiota structures. Within the family, each individual had a unique, personalized microbiota that allowed them to be differentiated from other members of their family. These personalized microbiota appear to develop at an early age, likely after weaning. Although it remains to be seen whether that is the microbiota that the children will carry with them through adolescence, it suggests that the differences in genetics and diet, environment, and life history characteristics imprint their effects on the microbiota at an early age. Despite the personalized nature of each microbiota, the overall family is clearly more similar to each other than they are to unrelated individuals from the broader community. Overall, these results confirm the model that individuals who share an environment likely share the same ecological meta-community that can colonize the microbiota and then be selected upon by host genetics, diet, and life history.

Although the microbiota of the family members are personalized to each member and are clearly distinct from those of the Ann Arbor community, there was still a large amount of temporal day-to-day variation. It is interesting that the underlying membership of each microbiota was consistent across the study for each person but the abundances of the individual populations were variable. Furthermore, we were unable to associate these fluctuations with diet, differences in environment, or health. This family experienced many disturbances to their microbiota via fluctuations in the composition of their diet and differences in environment. Yet their gut microbiotas were largely resilient to these disturbances. This suggests that the composition of their individual gut microbiota have been selected for to adapt to these disturbances. This suggests that adaptation by the microbiota to a personalized set of disturbances (for example, food preferences, hygiene, behaviors) helps to select for a personalized microbiota that is resilient to the disturbances.

The family considered in the current study will offer several opportunities to better understand the microbiota. First, our data suggest that the family represents a chronosequence, which can be used to understand the connection between child development and overall microbiota dynamics. For example, as the various children go through different life events such as weaning, puberty, and moving away from home, it will be possible to assess the effects of these events on the microbiota. Here, we saw the profound influence of complete weaning on the microbiota when viewing the 2-year-old who had not yet been weaned as a control for her older siblings. Second, this family offers the ability to better understand the effects of mode of birth on the development of the microbiota when controlling for genetics, environment, and diet, as the infant in this study was the only child to not be born vaginally. Following the development of the microbiota in this child relative to his siblings will help us to better understand the long-term impacts of Cesarean delivery. Finally, in the present analysis, the weaned children had similar microbiotas relative to their parents, suggesting that factors other than age or sex are most important in shaping their microbiota. Tracking these children to identify events that lead to deviations in microbiota structure will allow us to better understand the mechanisms that shape and reinforce the structure of these communities.

Families represent a special cultural entity with shared genetics, environment, diet, and microbiota. Unfortunately, they have been largely ignored as a medium for understanding how genetics, environment, and diet interact to form an individual’s personalized microbiota. All families are different and present different mixtures of genetics, environment, and diet. Although this family may be considered unique because of the large number of children in it, exposure to livestock, and homeschooling, all families have idiosyncrasies that make them unique. As our data suggest, children are born into an environment where they are provided with the family’s microbiota; however, their unique genetics, diet, and life history exert a selection on that microbiota to make their own at a very early age. Therefore, it is critical that we develop a better understanding of how individualized microbiota develop as a function of human social interactions with each other and their environment. How this translates to other communal living arrangements, such as establishing new families, dormitories, hospitals, and assisted living centers, is likely to yield a better understanding of the mechanisms that affect the structure and function of the microbiota.

## Abbreviations

BMI: body mass index; KEGG: Kyoto Encyclopedia of Genes and Genomes; KO: KEGG orthology categories; NMDS: non-metric dimensional scaling; OPF: operational protein family; ORFs: open reading frames; OTUs: operational taxonomic unit.

## Competing interests

The authors declare that they have no competing interests.

## Authors’ contributions

PDS and SJS designed the study and recruited the family which was analyzed in this study. PDS, KDI, JFP generated and analyzed the sequence data. All authors participated in the writing of the manuscript. All authors read and approved the final manuscript.

## Supplementary Material

Additional file 1 Diet record for family members. Record of food consumed by family members over the course of the study. Click here for file
